# Editorial: Cell structure and dynamics in the nematode *Caenorhabditis elegans*

**DOI:** 10.3389/fcell.2025.1669625

**Published:** 2025-08-11

**Authors:** Claudia Tanja Mierke

**Affiliations:** Biological Physics Division, Faculty of Physics and Earth System Sciences, Peter Debye Institute of Soft Matter Physics, Leipzig University, Leipzig, Germany

**Keywords:** *C. elegans*, actin cytoskeleton, tension, development, lifeact, myosin, transcriptional analysis, animal model system

The Research Topic “Cell structure and dynamics in the nematode *Caenorhabditis elegans*” emphasizes the importance of the classic animal model system *C. elegans* in the investigation of fundamental cell biological and biophysical questions. In addition to the continuous methodological measurement of the C. elegant animal model, mechanobiological aspects are increasingly coming to the foreground (Borne and Weiss, 2025). Moreover, the extracellular matrix (ECM) environment, such as matrix composition and/or mechanics (stiffness) can impact the lifespan of *C. elegans* (Teuscher et al., 2024). Classic analyses at the transcriptional level and molecular analyses are not being pushed into the background. They can also be combined in future studies to gain even deeper insights into the fundamental cell biological mechanisms and disease related mechanisms (Coraggio et al., 2024). The Research Topic deals with mechanobiological aspects such as tension on the actin cytoskeleton and apical cell connections of smooth muscle cells in *C. elegans*, and the role of TIAR1 in the formation of stress granules in *C. elegans*, transcriptomic analysis of oocyte formation during seven specific germline stages, the molecular mechanisms regulating the metabolic link to reproduction in *C. elegans*, and stress granules as well as experimental techniques for the use of Lifeact expression in *C. elegans*.

The Research Topic comprises five original experimental articles, including four original articles and a Brief Research Report. The first Original Research Article by Sadeghian et al. presents how the egg penetration-induced stretching of the spermatheca cells, an elastic, tubular tissue in the reproductive system of hermaphrodites and location of egg fertilization, influences the force distribution *in vivo* and how altered myosin activity leads to reactions in the spermatheca tissue in *C. elegans*. The authors carried out *in vivo* laser ablation studies to explore the actomyosin network and its dynamics. The authors show that a reduction in actomyosin contractility through deprivation of phospholipase C-ε/PLC-1 or non-muscular myosin II/NMY-1 causes bloated spermathecae, which are filled with one or several embryos. However, the tension on the basal actomyosin fibers was not affected. When myosin is activated by depletion of Rho GAP SPV-1, the tension on the actomyosin fibers is elevated, confirming previous findings that Rho drives the contractility of spermathecae. While the tension at the apical connections on the inner surface of the spermatheca tube is lowered by exhausting PLC-1 and NMY-1, the exhaustion of SPV-1 causes an elevation of basal contractility and the tension at the apical connections also declines, with the strongest effect seen at the junctions that are oriented vertically to the axis of the spermatheca. Ultimately, their findings contribute to expanding knowledge about the mechanical characteristics of the reproductive system and how cell structures and contractile proteins interfere with each other in controlling the mechanophenotype of spermathecae.

The second Original Research Article by Fuentes-Jiménez et al. describes how the formation of stress granules, which serve as storage of mRNA and thereby represses their translation, is regulated by key players, such as the two RNA-binding proteins TIA1 and TIAR1. Therefore, they broke two predicted α-helices inside the prion-like domain of the *C. elegans* TIA1/TIAR homolog TIAR-1 to determine whether its association with stress granules is critical for the threadworm. The researchers observe that under stress, tiar-1 PrD6 mutants still formed TIAR-1 condensates in the gonads of *C. elegans*, but the condensates were not properly formed or structurally organized. Nevertheless, TIAR-1 condensates seem unstable and disintegrated rapidly during stress. Stress granules appear to persist and fuse at regular intervals, as demonstrated by CGH-1, which serves as a marker for stress granules. In the same way as tiar-1 knockout worms, tiar-1 PrD6 mutants also experience fertility issues and a reduced lifespan. These results indicate that the proposed prion-like domain of TIAR-1 is critical for its linkage to stress granules. In addition, this domain could also perform important roles in several TIAR-1 activities, such as fertility, embryogenesis, and longevity, which are not related to stress, including oxidative stress or UV radiation. Consequently, the correlation between TIAR-1 and condensates is not relevant for the protection of the organism against stress or stress-induced apoptosis, suggesting that other molecules within the stress granule may perform this function.

The third Original Research Article by Su et al. explores the formation of oocytes within *C elegans*. In the past, the small size of the experimental animal and the challenges in obtaining specific stages of the germline have prevented a more detailed systematic investigation of this mechanism. The researchers pursue a different strategy basing their work on a transcriptomic analysis of *C. elegans* oogenesis. They dissect a hermaphroditic gonad into seven sections corresponding to the mitotic distal area, the pachytene area, the diplotene area, the early diakinesis area, and the three most proximal egg cells. The transcriptome of every single section was then sequenced along with that of the fertilized egg by means of a single-cell RNA sequencing. They figured out specific gene expression patterns and splicing events along the gonads and got new insights into the underpinnings of how eggs develop. In addition, they identified transcripts that may perform roles in interactions among the germ line and somatic gonadal cells. These results form the basis for future germline research, like on germline development, oogenesis, and fertilization events. Finally, the authors propose that applying a multi-omics approach to individual cells/stages of the gonads could provide a clearer picture of the regulation governing oogenesis.

The fourth Original Research Article by Breen et al. investigates the molecular mechanisms that control the linkage between metabolism and the reproductive systems in *C. elegans*. The researchers figured out that the previously unidentified *C. elegans* F-box protein FBXL-5 is part of the mTORC2 signaling route and helps to keep lipids in check. FBXL-5 thus acts as a negative regulatory factor in the maternal supply of vitellogenin lipoproteins, which facilitate the transport of intestinal lipids from the intestine to the germline. Mutation of fbxl-5 can partially suppress the vitellogenesis malfunctions seen in the lin-4 and lin-29 heterochronic mutants, each of which have ectopic expression of fbxl-5 in the adulthood stage. FBXL-5 negatively controls the expression of vitellogenin genes in the gut; so, having too much FBXL-5 in the gut is enough to impair vitellogenesis, limit lipid buildup, and reduce lifespan. Epistasis analyses indicate that fbxl-5 regulates vitellogenesis together with two other genes, the cul-6, a cullin gene, and the Skp1-related gene skr-3. In addition, fbxl-5 works genetically upstream of rict-1, which codes for the core protein Rictor of the mTORC2 complex, thereby controlling vitellogenesis. Collectively, these findings demonstrate an unprecedented involvement of an SCF-ubiquitin ligase complex in regulating lipid homeostasis in the intestine through activation of mTORC2 signal transduction. The research highlights a new mechanism that links metabolic control in the gut to a developmental timing signaling pathway.

The Brief Research Report by Ono investigates the effect of overexpression of the short peptide Lifeact in *C. elegans* on the body wall muscle, which is necessary for the body axis elongation in *C. elegans* embryos. Since mCherry Lifeact is a less invasive technique to monitor actin organization and remodeling, it is often used as a tool for visualizing actin filaments in live imaging. Ono hypothesizes that the binding site of Lifeact on F-actin, which intersects with the sites of binding for cofilin and myosin (Belyy et al., 2020), may influence the contractility of actomyosin. In addition, strong sarcomere disorder due to overexpression of Lifeact can impede muscle contraction and impair the development of embryos and larvae. In his fundamental work, Ono therefore investigates whether high concentrations of Lifeact can modify the dynamics of actin filaments and induce artificial alterations of the actin cytoskeleton by using *C. elegans* strains that express Lifeact fused to the fluorescent protein mCherry in the body wall muscle. He shows that low expression of Lifeact from a single transgene was sufficient to label sarcomeric actin filaments, but overexpression of Lifeact from an extrachromosomal array led to serious disruption of muscle sarcomeres and death at the embryo or larval phase. The expression of Lifeact must therefore be maintained at a low level through appropriate control of the expression system for imaging analyses of *C. elegans*.

The need for more research in the field of basic research and mechanobiology in combination with genetic, molecular, and helical biological analyses in this important animal model system remains. The bidirectional interactions of the animal model with the biological, biochemical, and mechanical properties of the microenvironment in physiological and pathological settings, such as cancer, also play a role. Finally, the refinement of established animal model systems such as *C. elegans* represents a promising strategy for future investigations of mechanobiological processes during development and disease.
